# Age-associated alterations in CD8α+ dendritic cells impair CD8 T-cell expansion in response to an intracellular bacterium

**DOI:** 10.1111/j.1474-9726.2012.00867.x

**Published:** 2012-12

**Authors:** Gang Li, Megan J Smithey, Brian D Rudd, Janko Nikolich-Žugich

**Affiliations:** Department of Immunobiology and the Arizona Center on Aging, University of Arizona College of MedicineTucson, AZ 85724, USA

**Keywords:** aging, cellular immunology, mice, T-cell

## Abstract

Age-associated decline in immunity to infection has been documented across multiple pathogens, yet the relative contributions of the aged priming environment and of lymphocyte-intrinsic defects remain unclear. To address the impact of the aging environment on T-cell priming, adult naïve OT-I TCR transgenic CD8 T cells, specific for the H-2K^b^-restricted immunodominant OVA_257-264_ epitope, were transferred into adult or old recipient mice infected with the recombinant intracellular bacterium *Listeria monocytogenes* carrying the chicken ovalbumin protein (Lm-OVA). We consistently found that adult OT-I CD8 expansion was reduced in aged recipient mice, and this correlated with numeric, phenotypic, and functional defects selectively affecting CD8α+ dendritic cells (DC). Following Lm-OVA infection, aged mice failed to accumulate CD8α+ DC in the spleen, and these cells expressed much lower levels of critical costimulatory molecules in the first three days following infection. Further, aged CD8α+ DC showed impaired uptake of the bacteria at very early time points following infection. Treatment of aged mice with Flt3 ligand (Flt3L) improved the number of DC present in the spleen prior to Lm-OVA infection, and improved, but did not reconstitute, OT-I expansion to Lm-OVA infection. These results suggest that age-associated changes in antigen uptake, pathogen sensing, and/or antigen presentation contribute to impaired adaptive immune responses to microbial pathogens with aging.

## Introduction

It is well established that older adults are more susceptible to many pathogens and respond poorly to vaccines. While various age-related impairments have been described in the innate and/or adaptive immune systems (reviewed in Haynes & Swain, 2012; Nikolich-Zugich *et al*., 2012; Solana *et al*., 2012), the relative importance of these defects remains incompletely understood and difficult to disentangle. Recently, we have shown that increased susceptibility to intracellular pathogens (i.e., West Nile virus, *Listeria monocytogenes*) in old mice correlates with numerically reduced and functionally impaired CD8 T-cell responses (Brien *et al*., 2009; Smithey *et al*., 2011). While these studies directly demonstrated the existence of a cell-autonomous age-related defect in T-cell immunity, contribution of defects in other cellular and molecular components of immunity that are important for T-cell triggering and differentiation was not examined and could not be excluded.

Understanding the totality of age-related changes in immunity, including delineation of the cell-intrinsic and environmental factors that contribute to suboptimal immune responses, is essential for developing optimal vaccines and immunomodulatory strategies for the aging population. In this study, we took advantage of the well-characterized *L. monocytogenes* (Lm) model system to study the effects of aging on both the early stages of DC infection by Lm, as well as the subsequent priming of CD8 T cells *in vivo*. The ability to mount robust CD8 T-cell responses against most pathogens depends upon efficient presentation of antigen by dendritic cells (DCs). In mice, up to six phenotypically distinct DC subsets have been categorized based on their expression of various surface markers (Shortman & Liu, 2002; Heath *et al*., 2004). Despite this heterogeneity, priming of CD8 T cells against intracellular viral and bacterial infections is mediated almost entirely by the CD8α+ dendritic cell subset (Belz *et al*., 2004; Campisi *et al*., 2011). Further, this subset plays a unique role early following intravenous infection with Lm, facilitating bacterial entry, early survival, and replication within the predominant tissues of infection (Neuenhahn *et al*., 2006; Campisi *et al*., 2011; Edelson *et al*., 2011; Mitchell *et al*., 2011; Waite *et al*., 2011). While DCs recovered from adult and old mice appear to have equal capacity to prime T cells *in vitro* (Moretto *et al*., 2008; Jiang *et al*., 2009), few studies have directly examined these responses in infected animals *in vivo*, within the host microenvironments where they occur.

We found that the early establishment of Lm infection within splenic CD8α+ DCs was impaired in old mice and that this population failed to accumulate in old mice later, on days 3–5 post-infection. Although the bacterial burden eventually reached equivalent levels in adult and old animals, the alteration of the early stages of infection correlated with a reduced or bimodal expression of costimulatory molecules on the CD8α+ DC subset in old mice. Moreover, lack of accumulation of splenic CD8α+ DCs later in the infection correlated to reduced capacity of transferred adult CD8 T cells to accumulate in old recipients. Both the number of CD8α+ DC and the magnitude of CD8 T-cell expansion following *Listeria* infection in old mice could be partially restored by treatment with Flt3L treatment. These studies reveal that, in addition to the observed cell-intrinsic defect in expansion and effector differentiation in aged CD8 T cells (Brien *et al*., 2009), both the very early and later host–pathogen interactions at the level of old CD8α+ DCs can have a further negative impact on successful generation of adaptive immune responses in aging organisms.

## Results

### Poor expansion of adult CD8 T cells in old recipients following Lm-OVA infection

**Fig. 1:**
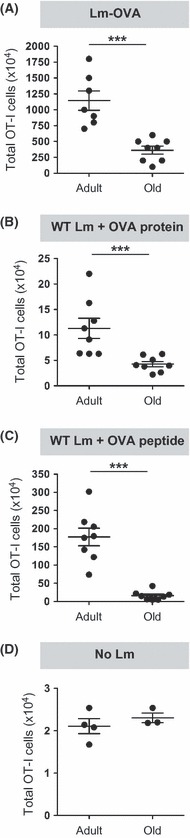
Adult OT-I CD8 T cells show reduced expansion in old mice in response to antigen stimulation. 5000 naïve CD45.1+ OT-I CD8 T cells were transferred into adult and old C57/B6 CD45.2+ recipients. The next day, animals were inoculated with (A) 10^4^ CFU Lm-OVA, (B) 10^4^ CFU wild-type Lm plus 100 μg OVA-8p peptide, or (C) 10^4^ CFU wild-type Lm plus 1-mg OVA protein. 7 days later, the number of OT-I CD8 T cells per spleen was determined. (D) 2 × 10^5^ Naïve CD45.1+ OT-I CD8 T cells were transferred into adult and old recipient mice and engraftment in the spleen was determined 24 h later. Data represent three independent experiments with (A–C) 7–8 mice/group, or (D) 4 mice group^−1^. ***P < 0.0001 by Student’s *t*-test.

The goal of this study was to determine the extent to which diminished CD8 T-cell responses previously observed in old mice (Brien *et al*., 2009; Smithey *et al*., 2011) may be further exacerbated by T-cell extrinsic factors in the aged host. Our general approach was to seed adult and old mice with equal numbers of identical adult OT-I TCR transgenic CD8 T cells and to compare their ability to expand in age-disparate recipient environments in response to microbial challenge. To this end, low numbers (5 × 10^3^) of congenically marked adult OT-I CD8 T cells (CD45.1+) specific for OVA-8p (SIINFEKL, the H-2K^b^-restricted ovalbumin epitope) were adoptively transferred into adult and old C57BL/6 recipients (CD45.2+). Following systemic infection with recombinant *L. monocytogenes* expressing the ovalbumin antigen (Lm-OVA), three to fourfold more OT-I cells were recovered in adult recipients relative to old (Fig. 1A).

It was possible that Lm-OVA may not produce the same amount of the OVA-8p epitope in adult and old hosts owing to any number of steps involved in early bacterial replication, and/or antigenic uptake, processing, and presentation. To control for such differences, we performed identical transfers but immunized the recipients with wild-type Lm (to keep the inflammatory context constant) admixed with OVA protein (eliminating any possible differences in Ag abundance) or the immunodominant SIINFEKL peptide (OVA-8p; eliminating differences in Ag abundance, uptake, and processing). The same three to fourfold difference was observed in each case, except that the absolute level of CD8 T-cell expansion was 100-fold lower with the OVA protein (Fig. 1B) and about 4–5× lower with the OVA-8p compared with Lm-OVA (Fig. 1C; note the difference in *Y* axis values in A–C), likely due to the much lower abundance of the epitope present when delivered outside the replicating pathogen. Another important control was to ensure that similar levels of engraftment of the transferred OT-I CD8 T cells occurred in the spleens of old and adult recipients. As it is technically very difficult to observe transferred cells when only 5 × 10^3^ cells are transferred (with an estimated take of 10–15%, this would yield ∼500–750 cells animal), we performed control experiments using the same cohort of recipients but with a transfer of 2 × 10^5^ OT-I cells into adult and old recipients. Analysis of the congenic CD45.1/CD45.2 marker system confirmed identical short-term OT-I cell engraftment in the spleen 24 h later (in the absence of infection), at the expected 10–15% level of the original inocula (Fig. 1D).

**Fig. 2:**
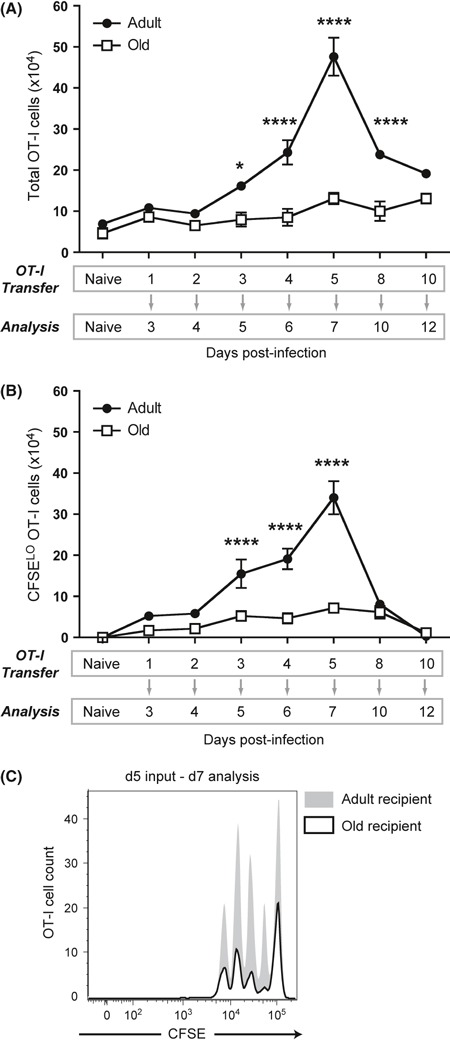
Old mice show reduced priming capacity throughout Lm-OVA infection. 2 × 10^6^ CFSE-labeled naïve CD45.1+ OT-I CD8 T cells were adoptively transferred into adult (closed circle) and old (open square) CD45.2+ recipients on the indicated days after infection with Lm-OVA. Two days later, (A) the total number of splenic OT-I CD8 T cells and (B) the number of CFSE^LO^ OT-I CD8 T cells determined. A representative OT-I CFSE profile of an adult (gray) and old (white) recipient from day 5 transfer is shown in (C). Note that all the mice were infected at the same time and that transfers were performed on different days, probably accounting for some of the variation in the total cell content over time in O mice from day 0 to day 12 in overall yields; nonetheless, expansion in these mice was clearly much reduced compared with the adult counterparts. Data represent two independent experiments with 4 mice group^−1^ time^−1^ point. *P < 0.05, ****P < 0.0001 by two-way anova with Bonferroni post-test.

Diminished adult CD8 T-cell responses in old mice could be owing to various T-cell extrinsic factors in the host. We therefore designed experiments to uncouple the stages of the immune response that contribute to the optimal expansion of CD8 T cells. While only a brief period of activation (24–48 h) is required to initiate proliferation of CD8 T cells (Kaech & Ahmed, 2001; van Stipdonk *et al*., 2001), sustained antigen presentation and inflammatory signals (>48 h) are required for robust CD8 T-cell expansion *in vivo* (Prlic *et al*., 2006). To investigate this question, we next transferred 2 × 10^6^ carboxyfluorescein methylester (CFSE)-labeled adult OT-I CD8 T cells into adult and old recipients at various time points following Lm-OVA infection, and examined dilution by these OT-I cells after 48 h *in vivo*. In this way, the CFSE-labeled OT-I CD8 T cells acted as indicator cells to examine the availability of antigen that can prime naïve T cells throughout the first 10 days following Lm-OVA infection in adult and old recipient environments. Both the total number of OT-I CD8 T cells (Fig. 2A) as well as the number of OT-I cells that had undergone at least one division (CFSE^LO^, Fig. 2B, with representative CFSE profiles in Fig. 2C) increased two to fourfold when transferred into adult mice between 3 and 5 days post-infection, suggesting a window of time during which conditions were optimal for expansion of naïve OT-I cells. There was relatively little expansion/accumulation of OT-I CD8 T cells in old recipients throughout the course of the infection (Fig. 2), suggesting that this window for expansion/accumulation, if present, is greatly reduced in aged animals. Although these results differ from previous work that observed a peak in ‘functional’ antigen display on days 1–2 days after *Listeria* infection (Porter & Harty, 2006), these prior experiments were performed with a reduced dose of *Listeria* and in the BALB/c genetic background which is 10-fold more sensitive to *Listeria* (reviewed in Garifulin & Boyartchuk, 2005), perhaps explaining the differences in kinetics. Our data suggest that the most robust accumulation of expanded T cells can be observed between 3–7 days following infection with higher *Listeria* infectious doses in the adult C57BL/6 mouse and that old mice have a reduced capacity to prime/expand/accumulate OT-I CD8 T cells during this critical window.

### Old mice fail to accumulate the CD8α+ DC required for efficient CD8 T-cell priming to *Listeria* at early time points

It has been well established that CD8 T-cell responses to *Listeria* infection are dependent on priming by CD11c+ DC (Jung *et al*., 2002). To confirm the importance of the CD11c+ DC population for antigen presentation following systemic Lm-OVA infection, we isolated total CD11c+ DC, B cells, and macrophages from Lm-OVA-infected adult mice on day 3 post-infection. Each purified antigen-presenting cell population was co-cultured *in vitro* with CFSE-labeled adult OT-I cells for 60 h to determine the priming capacity. We confirmed that only the CD11c+ DCs were able to induce division and CFSE dilution by adult OT-I cells (Fig. S1).

**Fig. 3:**
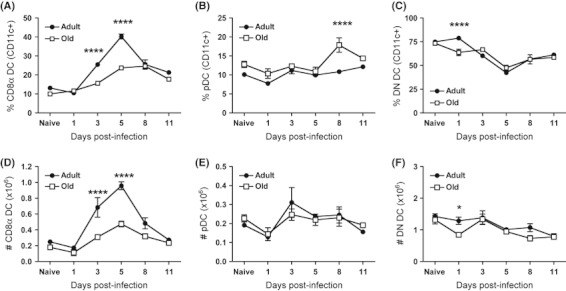
Old mice do not accumulate splenic CD8α+ DC following Lm-OVA infection. DCs were isolated from spleens of naïve and Lm-OVA-infected adult (closed circles) and old mice (open squares) at various time points, and the frequency (A–C) and total number (D–F) of CD8α+ DC (CD8α+ CD45RA−), plasmacytoid DC (CD8α− CD45RA+), and double-negative (DN) DC (CD8α− CD45RA−) cells was determined by flow cytometry. Data represent two independent experiments with 4 mice group^−1^ time point^−1^. *P < 0.05, ****P < 0.0001 by two-way anova with Bonferroni post-test.

The CD11c+ DC population can be further discriminated into (at least) three major DC subsets in the spleen based on the expression of CD8α and CD45RA: CD8α+ DC (CD8α+ CD45RA−), double-negative DC (CD8α− CD45RA−), and plasmacytoid DC (CD8α− CD45RA+) (Liu & Nussenzweig, 2010). Subsequent studies in the *Listeria* infection model have identified the CD8α+ DC subset as the cells responsible for efficient CD8 T-cell activation (Campisi *et al*., 2011; Edelson *et al*., 2011; Mitchell *et al*., 2011), as well as serving an important role in establishing a productive infection (Edelson *et al*., 2011; Mitchell *et al*., 2011; Waite *et al*., 2011). Therefore, we next determined the distribution of the total CD11c+ DC population into each of these subsets throughout the 11 days following Lm-OVA infection in adult and old mice (Fig. 3, with representative gating shown in Fig. S2). In both naïve mice, and animals evaluated one day after infection, there was no difference in either the frequency or total number of splenic CD8α+, double-negative (DN), or plasmacytoid (pDC) subsets. However, on days 3 and 5 following infection, there was a marked increase in the critical CD8α+ DC subset in adult animals that was notably absent in the old mice (Fig. 3A,D). This increase in the CD8α+ DC population in adult mice correlates with the time frame in which adult recipients of OT-I cells showed a greater capacity for CD8 T-cell expansion (Fig. 2). As previously shown (Neuenhahn *et al*., 2006; Edelson *et al*., 2011), when purified DC subsets from adult mice were co-cultured *ex vivo* 3 days after Lm-OVA infection, CD8α+ DC were the only cells able to stimulate OT-I division (Fig. S3). This highlights the potential functional importance of the inability to mobilize/accumulate this DC subset in aged mice on days 3–5 post-infection (Fig. 3A,D).

### Aged CD8α+ DC show poor upregulation of costimulatory molecules at early time points following Lm-OVA infection

**Fig. 4:**
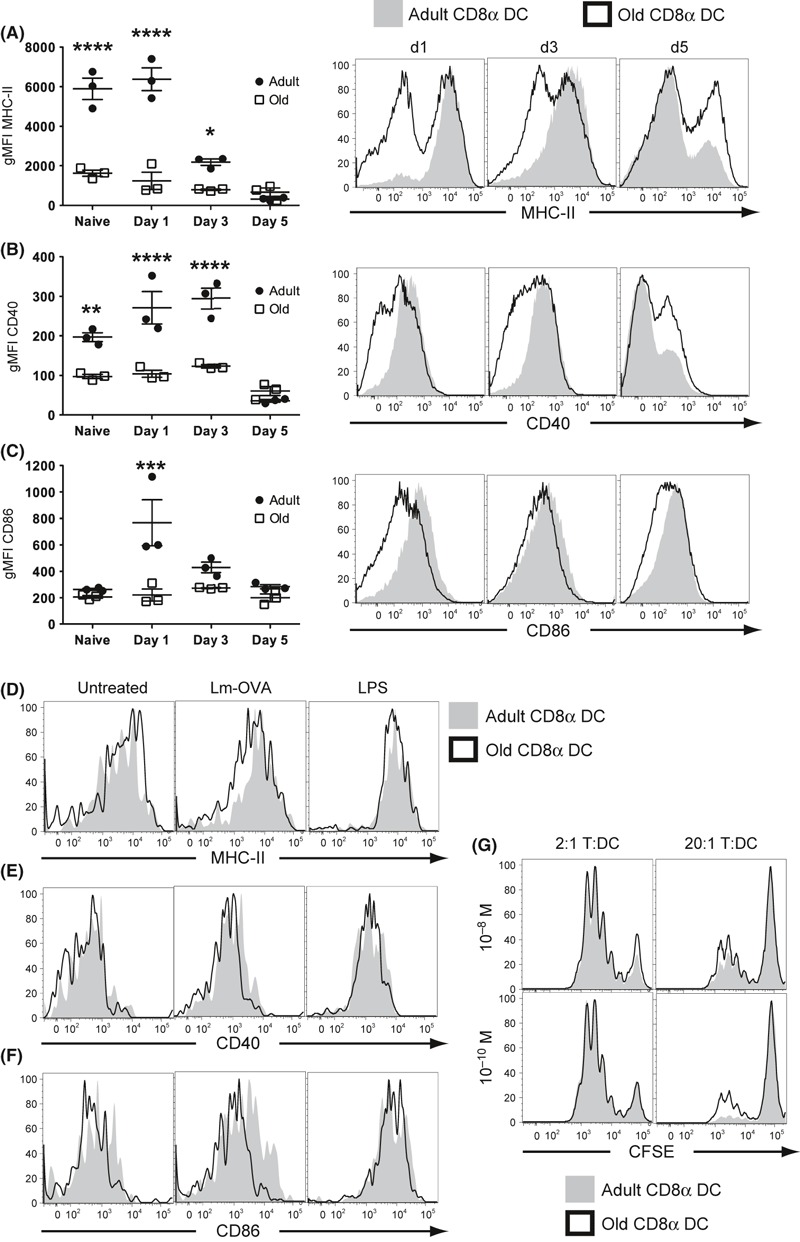
CD8α+ DC exhibit maturation defects *in vivo* following infection, yet function normally *in vitro*. (A–D) DCs were isolated from spleens of naïve and Lm-OVA-infected adult (closed circles) and old mice (open squares) at various time points, and the expression levels of (A) MHC-II, (B) CD40, and (C) CD86 were determined. Representative histograms of expression by adult (gray) and old (open-white) CD8α+ DC are shown. (D–F) CD11c+ DC from naïve adult (gray) or old (open/white histograms) spleens were purified and stimulated *in vitro* overnight with either Lm-OVA or LPS, and the upregulation within the CD8α+ DC subset of (D) MHC-II, (E) CD40, and (F) CD86 was determined 18 h later. (G) Adult (gray histograms) and old (white histograms) CD11c+ DC were stimulated overnight with LPS plus (upper row) 10^−8^ m or (lower row) 10^−10^ m SIINFEKL peptide, and then co-cultured with CFSE-labeled adult OT-I T cells at various T:DC ratios, as indicated, for 3 days. Data represent two independent experiments with 3 mice group^−1^ time point^−1^. *P < 0.05, **P < 0.01, ***P < 0.001 by two-way anova with Bonferroni post-test (A–C).

The ability to prime naïve CD8 T cells is critically dependent on the upregulation of various costimulatory molecules on the DC surface. Upregulation of CD40, CD80, and CD86 on CD8α+ DC has been shown to occur within 24 h of Lm infection in adult mice (Mitchell *et al*., 2011). To determine whether differences in costimulatory expression may be contributing to the impaired proliferation of adult OT-I cells in aged recipient mice (Figs. 1 and 2), the expression levels of MHC-II, CD40, and CD86 were measured on splenic DC subsets prior to and on days 1, 3, and 5 following infection with Lm-OVA. Higher overall basal expression of class II and CD40 was seen on resting CD8α+ DC in adult relative to old mice (Fig. 4A–C), with no differences in basal CD86 expression, as measured by geometric mean fluorescent intensity of these molecules. The age-related impaired upregulation of costimulatory molecules became more pronounced on day 1–3 post-infection, with old CD8α+ DC failing to upregulate any of them (Fig. 4A–C). Interestingly, when we evaluated the histograms for costimulatory expression, it was clear that adult CD8α+ DC appeared to almost uniformly upregulate these costimulatory molecules for the first 3 days post-infection. In contrast, old mice showed a bimodal expression pattern for both MHC-II and CD40, and failed to achieve uniform upregulation. For adult and old pDC and DN DC subsets (whose role in CD8 T-cell priming to Lm is less clear), differences were more modest. The pDC subset appeared to undergo greater maturation in old mice than young, while the response of DN DCs was relatively comparable (Fig. S4).

The question remained whether the differences in maturation seen between adult and old DC reflect an inherent defect in maturation and upregulation of costimulatory molecules, or whether this is a consequence of some breakdown or alteration of the *in vivo* infectious process in old animals. To address this, we next purified the total CD11c+ DC pool from adult and old näive mice and stimulated *in vitro* maturation with either Lm-OVA or LPS. As shown in Fig. 4D–F, incubation with viable Lm-OVA was mildly less effective at maturing old CD8α+ DC compared with adults *in vitro*. In contrast, there was no difference in the capacity of the CD8α+ DC to upregulate costimulatory molecules following *in vitro* stimulation with LPS. Further, when LPS-matured adult and old DC were pulsed with SIINFEKL peptide and co-cultured with adult OT-I cells, equivalent OT-I proliferation was observed regardless of T/DC ratio or peptide concentration (Fig. 4G). Collectively, these experiments show that old DC can undergo efficient maturation in response to an ‘easy’ maturation signal *in vitro* (free LPS), but may be less capable of detecting and responding to a maturation signal provided by an intact pathogen/infection. Thus, age-related defects in CD8α+ DC recognition of pathogen and the subsequent upregulation of costimulatory molecules may have downstream consequences on the priming and/or accumulation of naïve CD8 T cells in the aged environment.

### Aged mice poorly establish productive Lm-OVA infection within CD8α+ DC

**Fig. 5:**
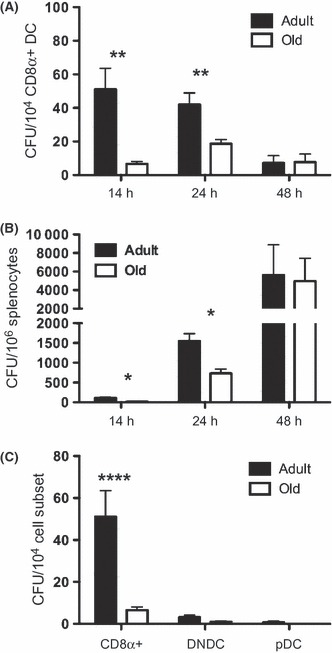
Old DC show decreased uptake of Lm-OVA at early time points following infection. Adult (closed bars) and old mice (open bars) were infected with 1 × 10^5^ CFU Lm-OVA and the number of intracellular bacteria within the (A) CD8α+ DC or (B) total splenocyte population was determined by cell sorting at various time points post-infection. (C) At 14 h post-infection, the intracellular Lm burden within different splenic DC subsets was determined. Data represent two independent experiments with 4 mice group^−1^ time point^−1^. *P < 0.05, **P < 0.01, ****P < 0.0001 by two-way anova with Bonferroni post-test.

Within hours of Lm infection, those bacteria that escape neutrophil killing in the spleen are almost uniquely found within the CD8α+ DC subset (Neuenhahn & Busch, 2007; Waite *et al*., 2011). In mice lacking this subset, productive infection with *Listeria* is severely diminished (Edelson *et al*., 2011). We have previously reported an overall decrease in the bacterial burden within the spleen of old mice relative to adults at 24 h post-infection, although this difference was no longer apparent by day 3 (Smithey *et al*., 2011). Although we found no differences in the number of CD8α+ DC in the spleen of uninfected aged mice (Fig. 3D), we wondered whether their uptake of bacteria from the bloodstream, and therefore the early kinetics of establishment of infection, might be altered. To address this, the CD8α+, DN DC and pDC subsets were sorted from the spleens of adult and old mice infected 14, 24 or 48 h previously with Lm-OVA (Fig. 5). At 14- and 24-hr post-infection, the number of bacteria within the CD8α+ DC subset (Fig. 5A), as well as within the unfractionated total splenocyte population (Fig. 5B), was significantly reduced (10-fold at 12 h) in aged mice, whereas, by 48 h post-infection, bacterial burdens were similar, suggesting robust replication of Lm in both old and adult DC. There were very few bacteria recovered from other DC subsets at 14 h post-infection, suggesting that the pattern of infection remains the same in aged mice, with CD8α+ DC being the primary reservoir of viable intracellular bacteria (Fig. 5C).

Collectively, our data indicate that initial bacterial uptake by this specialized cell subset is much less efficient in aged animals. This change in the early dynamics of infection in aged mice, with fewer bacteria present during the first 24 h, correlates with decreased and/or bimodal upregulation of costimulatory molecules by the CD8α+ DC population in aged animals at early time points post-infection (Fig. 4A–C), and with decreased CD8 T-cell priming and accumulation (Figs. 1 and 2).

### Flt3 ligand treatment of old mice improves OT-I priming to Listeria

**Fig. 6:**
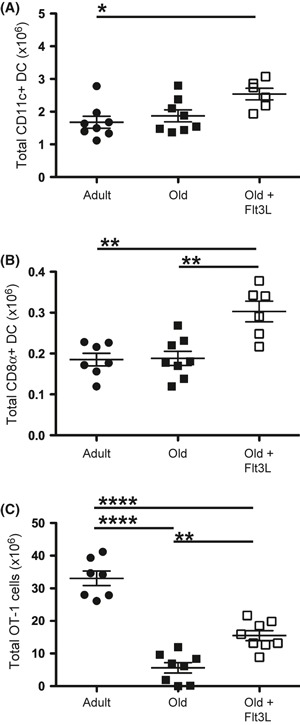
Flt3L treatment increases DC numbers and improves OT-I priming in old mice. Old mice received 10 μg rh-Flt3L intraperitoneally for seven consecutive days (open squares), and the number of (A) total CD11c+ DC or (B) CD8α+ DC in the spleen was determined relative to untreated adult (closed circles) and old mice (closed squares). (C) Adult, old, and old mice treated with Flt3L received 5000 OT-I cells, followed by Lm-OVA challenge, and the number of OT-I CD8 T cells was determined 7 days later. Data represent two independent experiments with 8 mice group^−1^ time point^−1^. *P < 0.05, **P < 0.01, ***P < 0.001 by one-way anova with Bonferroni post-test.

DC development is dependent on the growth factor Flt3L, and exogenous administration has been shown to both increase the number of CD8α+ DC in the spleen of young mice (Maraskovsky *et al*., 1996; O’Keeffe *et al*., 2003) and the bacterial burden in the spleen following infection with Lm (Alaniz *et al*., 2004). To evaluate whether old DC populations could be similarly expanded *in vivo*, old mice were treated for seven consecutive days with recombinant human Flt3L, and then the number of total CD11c+ DC as well as the CD8α+ DC subset in the spleen was determined. Although Flt3L treatment had a very modest effect on the number of total CD11c+ DC (Fig. 6A), it had a highly significant effect on the CD8α+ DC subset in old mice (Fig. 6B). To determine whether this improvement in CD8α+ DC numbers might improve the ability of the old environment to support OT-I proliferation after Lm-OVA infection, adult and old untreated mice as well as old mice treated with Flt3L underwent OT-I adoptive transfer followed by Lm-OVA infection. Seven days later, the number of OT-I cells recovered from Flt3L-treated old mice was significantly improved, albeit the number never approached that found in adults (Fig. 6C). These results suggest that increasing the overall DC population in old mice can improve their ability to effectively stimulate CD8 T cells. Limitations of this intervention and the implications of this result for our understanding of the relative importance of age-related defects in DC subsets are discussed below.

## Discussion

We have previously reported impaired T-cell priming in aged mice infected with either West Nile virus (WNV) or Lm-OVA (Brien *et al*., 2009; Smithey *et al*., 2011), and for WNV our experiments clearly documented the importance of T-cell-intrinsic age-related defects. This work sought to clarify the contributions of the aged environment over and above the cell-intrinsic problems within the aged T cells. Our data are consistent with a model in which CD8α+ DC serve two inter-related roles in the immune response to systemic *Listeria* infection, in such that the ability to prime an effective CD8 T-cell population to mediate Lm clearance is dependent on early and efficient establishment of infection within the CD8α+ DC subset.

What is the relationship between the very early events following infection and the subsequent ability to generate a robust T-cell response to the pathogen? Numerous reports have demonstrated the importance of the CD8α+ DC subset in the establishment of Lm infection in the spleen following i.v. inoculation of mice (Neuenhahn *et al*., 2006; Neuenhahn & Busch, 2007; Campisi *et al*., 2011; Edelson *et al*., 2011; Mitchell *et al*., 2011; Waite *et al*., 2011). How does this relate to the dominant role for this same subset in priming the CD8 T-cell response required for clearance of the bacteria? We propose that the inefficient priming of adult OT-I CD8 T cells in aged recipient mice is a function of at least two processes: (i) early problems with the uptake of Lm by the CD8α+ DC subset; and (ii) the concurrent problem in mobilization of these cells from DC precursors, as evidenced by the failure of old mice to increase the number of CD8α+ DC cells following Lm-OVA infection.

In adult animals, Lm escapes the phagosome rapidly, with ∼50% of the bacteria within the CD8α+ DC subset located in the cytosol within 6 h following i.v. infection (Muraille *et al*., 2007). It is presumed that intracellular bacterial replication provides a supply of *Listeria-*derived antigens for the class I MHC antigen presentation pathway, while bacterial by-products trigger multiple innate pathogen-recognition pathways (reviewed in Eitel *et al*., 2011). It is required that Lm escapes the phagosome and enters into the cytosol for efficient CD8 T-cell priming (Muraille *et al*., 2007). In theory, injection of either heat-killed Lm or strains incapable of escaping the phagosome should activate pathogen-recognition receptors, while bacterially derived products would be delivered to CD8α+ DC for cross-presentation to T cells. Yet, neither of these vaccination regimens leads to effective priming of protective T-cell populations *in vivo* (Lauvau, 2001), and in fact, Lm strains unable to escape the phagosome actively suppress the development of T-cell responses to cytosolically located bacterial antigens (Bahjat *et al*., 2009).

Thus, Lm infection within the cytosol of the CD8α+ DC population appears to be a key component of successful CD8 T-cell priming to this pathogen. Further, infected DC are relatively inefficient at killing intruding pathogens (Nagl *et al*., 2002; Alaniz *et al*., 2004), suggesting that productive infection of these cells, and their presentation of antigen from actively replicating intracellular pathogens, may be an important process in facilitating adaptive immune responses in general. Similar to infection with Lm, it has been shown that *ex vivo* antigen presentation by DC harvested 24 or 48 h following either subcutaneous or intravenous infection with HSV-1, influenza, or vaccinia virus infection is also limited to the CD8α+ DC subset (Belz *et al*., 2004), although the roles of direct infection vs. cross-presentation in these studies were not separated. Xu *et al*. (2010) have elegantly demonstrated the importance of direct priming, as opposed to cross-presentation or cross-dressing, by vaccinia-infected antigen-presenting cells as critical for stimulating CD8 T-cell responses, although that work did not classify which DC subset was responsible. Whether direct targeting of pathogens to the CD8α+ DC subset is a common mechanism for eliciting adaptive immunity to intracellular pathogens remains unclear.

The efficiency of pathogen-sensing processes within aged DC is in the early stages of investigation. Following murine infection with influenza, aged DC show impairments in NLRP3 inflammasome/caspase-1 activation, and downstream IL-1β and IL-18 production, suggesting age-related impairments in DC pathogen sensing and/or innate activation (Stout-Delgado *et al*., 2008). In humans, similar problems are seen in aged DC differentiated from PBMCs, including reduced expression of many toll-like receptors (TLR), impaired IFN-I production following TLR stimulation, and alterations to the downstream IFN-I signaling response following exposure to TLR ligands (Qian *et al*., 2011). Following oral infection of aged mice with *Endozoon cuniculi* microsporidia, Moretto *et al*. (2008) observed age-related T-cell impairments in conjunction with defects in DC within the mesenteric lymph nodes. These studies demonstrated that aged DC cultured with *E. cuniculi* were impaired in their ability to produce either IL-12 or IL-15, and showed poor upregulation of costimulatory molecules. Our preliminary studies found no difference in IL-12 production by DC when evaluated *ex vivo* at early time points following Lm-OVA infection of adult and old mice (data not shown); however, these may simply reflect differences in the specific pathogens investigated, *in vivo* (this study) vs. *in vitro* (Moretto *et al*., 2008) infection of DC, as well as the timing of the experimental evaluation.

Pathogen sensing may not be the only problem in aged mice, and it is likely that efficient delivery of Lm into the CD8α+ DC subset as a result of age-related changes within the splenic environment may also compromise the establishment of infection. Brown *et al*. (2011) have demonstrated that changes to the splenic marginal zone architecture of aged mice have profound consequences for the development of prion disease after administration of scrapie. In this model system, i.v. injection of scrapie leads to uptake of complement-coated complexes within the marginal zone, and then transfer to follicular DC, where accumulation precedes neuroinvasion. Aged mice do not develop disease at same incidence as young animals, and this correlates with a failure to accumulate scrapie agent in the folicular DC areas of the splenic architecture. Immunohistochemical analysis revealed that the architecture of the marginal zone (where particulate antigens are filtered from the blood) is disrupted in aged mice, and the ability to trap pre-formed, IV injected complexes was compromised.

We are aware of one report of impaired phagocytosis by aged monocyte-derived DC isolated from blood samples from elderly human subjects (Agrawal *et al*., 2007), but to our knowledge ours is the first to suggest *in vivo* age-related phagocytosis defects in lymphoid DC. Whether our data truly reflect specific defects in Lm uptake by aged CD8α+ DC or rather are a consequence of age-related changes to the splenic marginal zone architecture that compromise filtration of particulate antigens from the bloodstream will require further clarification. The former explanation is supported by the fact that a similar strategy of Flt3L pre-treatment prior to Lm infection of adult mice was found to both increase the number of DC and cause greater bacterial burdens and mortality, even in the presence of a robust, protective CD8 T-cell response (Alaniz *et al*., 2004). Unfortunately, these experiments did not clarify whether treatment with Flt3L led to more DC being infected by Lm, or to higher bacterial numbers within individual cells. As the mechanism underlying Lm targeting into the CD8α+ DC has been recently demonstrated to depend on serum complement proteins and platelets (Edelson *et al*., 2011), such mechanistic studies are underway. Experiments to distinguish between these possibilities and to elucidate whether similar DC defects also further disadvantage T-cell responses to other intracellular infections in old organisms are currently under investigation.

Our data suggest that poor establishment of Lm-OVA infection within the CD8α+ DC subset of aged mice within the first 24 h of infection (Fig. 5), and the lower costimulatory molecule expression by this subset over the first 3 days of infection (Fig. 4), may set up a less effective T-cell priming environment in old mice. This can be partially overcome by increasing the DC population in old mice by Flt3L treatment prior to infection (Fig. 6). However, it is also clear that this numeric increase in the DC available to take up Lm cannot truly compensate to increase T-cell priming, likely because the efficiency of bacterial uptake into individual CD8α+ DC remains compromised. Alternatively, there may be issues of DC recruitment that we have not been able to clearly evaluate owing to the lack of commercially available aged congenic mice for bone marrow chimera studies. The relationship between the nearly immediate problem of targeting Lm delivery to the splenic CD8α DC subset in old mice (Fig. 5), then problems in recruiting additional CD8α+ DC to the spleen in the first few days post-infection (Fig. 3), and the inability to upregulate costimulatory molecules on those CD8α+ DC are not entirely reconciled. Our OT-I transfer experiments indicate that the ideal conditions for T-cell priming are found somewhere between days 3–7, whereas the various defects we observed within the old CD8α+ DC subset occurred between 14 h and 3 days post-infection. One interpretation is that the use of adult OT-I transfers as a tool to detect ‘optimal’ antigen presentation conditions is somewhat misleading in that we may be measuring an environment that is above and beyond the conditions that are required for T-cell priming in an unperturbed system. Alternatively, these results may signal larger age-related problems affecting multiple arms of the DC populations that have not been previously appreciated.

The reduced capacity of the aged environment to prime adult OT-I CD8 T cells is reminiscent of truncated T-cell expansion seen when Lm infection is aborted early using either toxin-mediated depletion of CD11c+ DC (Prlic *et al*., 2006) or via antibiotic treatment (Porter & Harty, 2006). In contrast, antigen hyper-secreting strains of Lm appear to stimulate normal CD8 T-cell responses following early antibiotic treatment, suggesting both early events within the CD8α+ DC population as well as antigen density contribute to the magnitude of the response that develops (Smithey *et al*., 2008). Old mice do eventually reach the same CFU burden as adults as the Lm infection progresses (Fig. 5), and (presumably) antigen availability eventually catches up. Nonetheless, this is insufficient to overcome diminished early T-cell stimulation observed in the aged priming environment.

Defects in DC uptake of Lm, in reduced MHC and costimulatory molecule expression, and in accumulation of the critical CD8α+ DC subset at discrete times post-infection, provide another piece of the puzzle explaining why aged mammals fail to mount robust CD8 T-cell responses to *Listeria*. We conclude that both CD8 T-cell-intrinsic and environmental/antigen presentation issues contribute to impaired adaptive immune responses against intracellular bacterial pathogens in aged mice and suggest targeted manipulations to correct this defect. How alterations of CD8α+ DC function in aged organisms may influence adaptive immunity to other pathogens still remain to be fully elucidated.

## Experimental procedures

### Ethics statement

All animal research was conducted in strict accordance with the recommendations in the Guide for the Care and Use of Laboratory Animals of the National Institutes of Health. The studies were approved by the University of Arizona IACUC under the protocols #09-005 and subsequently #08-102. No human research was performed in this study.

### Mice

Old (18 and 22 months) C57BL/6 (B6, H-2b) mice were obtained from the National Institute of Aging breeding colony (Harlan). Adult (2–3 months) B6 mice were purchased from Jackson Laboratory (Bar Harbor, ME, USA). OT-I TCR transgenic CD45.1+ mice were bred in our existing specific pathogen-free barrier colony. All mice were maintained under specific pathogen-free conditions in the animal facility at the University of Arizona and experiments were conducted under guidelines set by the University of Arizona Institutional Animal Care and Use Committee.

### *Listeria monocytogenes* infections

Mice were systemically infected by intravenous injection in the lateral tail vein with either ‘wild-type’*L. monocytogenes* [strain 10403 originally obtained from Dr. David Hinrichs, Portland VA Medical Center, OR (Smithey *et al*., 2008)] or recombinant *L. monocytogenes* expressing the ovalbumin protein [Lm-OVA, provided by Dr. Hao Shen, University of Pennsylvania, PA (Pope *et al*., 2001)] in a volume of 100-μl sterile PBS. Unless otherwise stated, mice received 1–3 × 10^4^ colony-forming units (CFU) of *Listeria*. For all experiments, the number of inoculated bacteria was determined retrospectively by plating serial dilutions of the injected bacterial suspension onto brain-heart infusion (BHI) agar and counting colonies the next day.

### Flow cytometry and reagents

mAbs anti-CD11c (clone N418), anti-CD8α (53-6.7), anti-CD3 (500A2), anti-CD45RA/B220 (RA3-6B2), anti-CD45.1 (A20), anti-CD45.2 (104), anti-CD4 (RM4-5), anti-CD19 (eBio1D3), F4/80, anti-pan-NK (DX5), anti-MHC class II (M5/114.15.2), anti-CD40 (1C10), anti-CD80 (16-10A1), and anti-CD86 (GL1) were purchased from eBioscience, BioLegend, or BD Biosciences. Cells were stained 30 min to overnight at 4 °C with fluorochrome-conjugated antibodies specific for the surface markers. Cytofluorometric data acquisition was performed on a custom-made, four-laser BD Fortessa flow cytometer (Becton Dickinson, Sunnyvale, CA, USA) and was analyzed using FlowJo software (Tree Star, Inc., Ashland, OR, USA).

### Isolation of DC and other cell populations from spleens

Spleens were digested with Accutase (eBioscience, San Diego, CA, USA) for 30 min at 37 °C, and then cells were passed through a 40-μm mesh screen to prepare single-cell suspensions for analysis. CD11c^+^ cells were enriched by AutoMACS (Magnetically Activated Cell Sorter; Miltenyi Biotec, Bergisch Gladbach, Germany) before sorting into specific subsets by fluorescence-activated cell sorting (FACSAria, Becton Dickinson). Macrophage and B cells were isolated with CD19 or CD11b microbeads by AutoMACS. CD8 T cells were enriched by AutoMACS without digestion of spleen.

### CFSE labeling

OT-I splenocytes were resuspended at 10^8^/mL in PBS +0.1% BSA, and then mixed 1:1 with a 10-μm working solution of CFSE. Cells were incubated in the dark for 10 min at 37 °C, and then 5× the staining volume of ice-cold RPMI + 5% fetal calf serum (FCS) was added. Cells were incubated 5 min on ice, and then washed 3× more with RPMI + 5% FCS before used.

### *In vitro* dendritic cells maturation and OT-I proliferation

CD11c+ DCs were isolated from adult and old mice as above, and graded numbers were seeded in 100 μL RPMI 1640 + 10% FCS in 96-well U-bottom plates. DC were stimulated overnight with either 0.1 μg mL^−1^ LPS (Sigma, St. Louis, MO, USA) or Lm-OVA at an MOI of 1. Costimulatory molecule expression on DC subsets was determined by flow cytometry. For OT-I proliferation, DC stimulated overnight with LPS were then pulsed for 5 h with SIINFEKL peptide. DC were washed twice; then, 5 × 10^4^ enriched CFSE-labeled CD45.1+ CD8+ OT-I TCR transgenic cells were added a T:DC ratios of 2:1 to 20:1. Cultures were analyzed for proliferation after 60 h.

### Analysis of antigen presentation *in vivo*

Mice were inoculated with Lm-OVA, and then at various time points post-infection; 2 × 10^6^ CFSE-labeled OT-I TCR transgenic cells were adoptively transferred by intravenous injection into *Listeria*-infected or control recipients. At indicated days post-transfer, the proliferation of CFSE-labeled OT-I cells in the spleen was determined by flow cytometry. The transferred donor OT-I and endogenous recipient T cells were distinguished by flow cytometric detection of the CD45.2 molecule.

### *Ex vivo* sorting of *Listeria*-infected dentric cells subsets

To enumerate intracellular bacteria within different cell subsets at early time points post-infection, spleens were processed through CD11c+ enrichment as above with all steps performed in the presence of 5 μg mL^−1^ gentamycin to kill extracellular bacteria. Cells were incubated for 45 min on ice with anti-CD11c, CD11b, CD8α, CD45RA/B220 antibodies to discriminate cell subsets, and CD3, CD19, and DX5 antibodies in a ‘dump gate’ to exclude contaminating B, T, and NK cells. Cell subsets were then sorted on a FACSAria cell sorter. 3000–300 00 cells of each DC subset were sorted into pure FCS. Cell purity was confirmed by flow cytometry and found to be > 95%. Cells were washed in sterile PBS, and then lysed in sterile water before plating serial dilutions onto BHI agar. CFU were enumerated after incubation at 37 °C for 24 h.

### Treatment of old mice with Flt3 ligand

Old mice were injected intraperitoneally for seven consecutive days with 10 μg recombinant human Flt3 ligand (eBioscience) in 100 μL LPS-free PBS. The next day, mice were infected with Lm-OVA.

### Statistical analysis

Data are expressed as the mean ± SEM, and a representative experiment (of 2–4 repeats) is shown for each figure. All statistical analyses were performed by either Student’s *t*-test or two-way anova with Bonferroni post-tests using Prism software (GraphPad Software Inc., San Diego, CA, USA). Probability values of *P* < 0.05 were considered to be significant. The following notations have been used to denote *p* values in all figures: *P < 0.05; **P < 0.01; ***P < 0.001, ****P < 0.0001.

## Supporting Information

**Fig. S1** Only CD11c+ DC from infected mice stimulate OT-I division *ex vivo*.

**Fig. S2** Representative gating strategy for identification of CD11c+ dendritic cell subsets.

**Fig. S3** Only CD8α+ DC from infected micestimulate OT-I division *ex vivo*.

**Fig. S4** Costimulatory molecule expression on DN DC and pDC following Lm-OVA infection.
